# Adolescent criminality: multiple adverse health outcomes and mortality pattern in Swedish men

**DOI:** 10.1186/s12889-019-6662-z

**Published:** 2019-04-11

**Authors:** Marlene Stenbacka, Tomas Moberg, Jussi Jokinen

**Affiliations:** 1 Department of Clinical Neuroscience/Psychiatry, Karolinska Institutet, Stockholm, Sweden; 2 Department of Clinical Sciences, Psychiatry, Umeå University, Umeå, Sweden

**Keywords:** Alcohol, Criminality, Violence, Substance use, Mortality

## Abstract

**Background:**

To investigate the impact of adolescent violent and non-violent criminality and subsequent risk of morbidity and mortality in adulthood in a large Swedish cohort of young men conscripted for military service in 1969/70.

**Methods:**

The cohort consisted of 49,398 18-year-old Swedish conscripts followed up for morbidity and mortality up to the age of 55 years in Swedish national registers. Information about convictions for crime before conscription was obtained from national crime registers. Data from a survey at conscription were scrutinized to get information on potential confounders.

**Results:**

Hospitalization due to alcohol and drug related diagnoses and attempted suicide were significantly more evident in the violent group compared to non-violent criminals and non-criminals. More than one fifth (21.13%) of the young violent offenders, 12.90% of the non-violent offenders and 4.96% of the non-criminals had died during the follow-up period. In Cox proportional multivariate analyses, young violent offenders had twice the hazard (HR = 4.29) of all-cause mortality than the non-violent offenders (HR = 2.16) during the follow-up period. Alcohol and drug related mortality, suicide and fatal accidents were most evident in both violent and non-violent offenders.

**Conclusions:**

Men with adolescent criminality received more inpatient care due to alcohol and drug related diagnoses and attempted suicide as adults. Mortality due to unnatural causes, alcohol, and drug related diagnoses, suicide and accidents was most evident in violent offenders, while these causes of death were much lower in non-criminals. Men with adolescent criminality are a high-risk group for multiple adverse health outcomes and for early death. Efforts for detection of substance use and psychiatric disorders in this group is important for the prevention work in both local- and community levels as well as national prevention programs.

## Background

Criminality at young ages is associated with adverse negative outcomes, such as excess mortality including suicide and accidents as well as death by natural causes [1–6]. A great part of young criminals come from disadvantage environments which probably increase the risk of ill-health and premature death [7]. Violent offenders are often more troubled with e.g. conduct problems, low self-control, low intelligence, mental illness and alcohol and substance use compared to non-violent criminals [8–11]. Additionally, comorbid substance use behaviors and mental illness are common among criminals, which complicate both rehabilitation and treatment [9]. These individuals more frequently experience multiple health, social and behavioral problems and relapse more often in both hospitalizations and adult criminality, making this group more vulnerable to long-term negative outcomes including premature death [12–14]. It has been shown that early debut with criminality and continuous criminality in adulthood are associated with increased death rate and hospitalization compared to those young people who show criminal behavior only during adolescence [15].

In a clinical study of 1400 child and adolescent psychiatric patients (CAP), it was found that those who had been convicted during or prior CAP care were more often admitted to adult psychiatric care due to conduct problems than those not convicted; 34 vs. 13%. Likewise, adult substance related diagnoses were more evident in the conviction group than among non-criminals; 21 vs. 2% [16]. Offenders with substance use disorders benefit often from substance use rehabilitation and treatment with reduced crime rates as a positive outcome [17], showing that drug use and crime are often closely related to each other [16]. Likewise, it has also been found that hospital stays and hospital days in psychiatric patients decrease during the time in rehabilitation community programs compared to the time before enrollment [12]. These clinical studies demonstrate the importance of prevention programs in order to reduce criminality in people with comorbidity.

Few studies have investigated the association between early crime, both violent and non-violent, and hospital treatment for any diagnoses. The existing studies addressing the relationship between crime and hospitalization have focused on the early hospitalization in psychiatric diagnosis and later criminality [18]. Thus, in this study, we investigated whether adolescent criminality, violent and non-violent, was associated with more inpatient care stays and higher mortality rate compared to adolescents without criminal history in a large cohort of 49,398 18–20 years old men conscripted in 1969/70 for military service in in Sweden during a 37-years follow-up period. Another aim was to analyze the risk for causes of specific and overall mortality in the three criminal groups adjusted for relevant confounders for the outcomes. We anticipated observing especially increased risks of inpatient care stays and death among men debuting with violent crimes at adolescence versus the two reference categories: men with early debut of non-violent crimes and men without a history of adolescent criminality.

## Methods

The study is based on a cohort of 49,433 men who were compulsory conscripted to military service between 1 July in 1969 and 30 June in 1970. Due to psychiatric or physical handicap, about 2 to 3% were exempted. The study population and descriptions of methods including registry data of crime convictions, hospital care and mortality have been described in detail in earlier publications from our research constellation [1, 11, 19, 20].

### Criminal records

To identify date, type, and number of criminal offense before conscription, we used data from the national Crime Register containing detailed information on all convictions from 1966 onwards in Sweden. We used following criminal offenses to define serious violent crime (*n* = 196): conviction for homicide, manslaughter, aggravated assault, assault and battery, bodily harm and other. Further, we applied a dichotomization of violent offenses: conviction for violence (none vs. at least one).

### Hospital data

We used the National Hospital Register data for inpatient care for attempted suicide and alcohol and drug use diagnoses according to International Classification of Diseases (ICD) ICD-8, ICD-9 (between1987–1996) and ICD-10 (1997-). The National Hospital Register has detailed information of inpatient care. It reached first a full coverage of all public hospitals in Stockholm and Uppsala County in 1972, and since 1987, it covers 98–99% of inpatient care stays in Sweden.

### Mortality data

To investigate mortality and causes of death, we used the Cause of Death Register data. The Cause of Death Register, based on information from death certificates, covers more than 99% of all deaths occurring in Sweden. Underlying causes of death (one underlying cause of death is given on each death certificate, although contributing causes can be added), are classified according to ICD-8, ICD-9 and ICD-10. We used the following ICD classifications and codes for hospitalization and mortality data. Substance use disorders: Alcohol misuse - ICD-8: 291, 303, 571.00, 571.01 and 980; ICD-9: 291, 303, 305A, 357 F, 425 F, 535D, 571A-571D and 980; and ICD-10: E24.4, F10, G31.2, G62.1, G72.1, I42.6, K29.2, K70, K86.0, O35.4, P04.3, Q86.0, T51, X45, Y91, Z50.2 and Z71.4. Drug misuse - ICD-8: 304 and 965.0; ICD-9: 304, 965A, 968 F, 969 G and 969H; and ICD-10: F11–12, F14, F15, F16, F18, F19, O35.5, P04.4, T40.0-T40.3, T40.5-T40.9, T43.6, Z71.5, and X42. Suicide or suicide attempt were classified according to ICD-8 and ICD-9: E950-E959 and ICD-10: X60-X84 or as suicide with undetermined intent ICD-8 and ICD-9: E980-E989 and ICD-10: Y10-Y34. Circulatory diseases: ICD-8 and ICD-9: 390–459, ICD 10: I00-I99. Neoplasm: ICD-8 or ICD-9: 140–239, ICD-10: C00-D48. [1]

### Potential confounders

At conscription, each subject had to fill out two questionnaires. The first contained questions about family history, psychological and physical conditions. The second was mostly dealing with alcohol and substance use [21]. Based on the results from interviews and the questionnaires military psychologist assessed the ratings on a 9-point Likert scale, which was then collapsed to a 5-point scale measuring emotional control (measures of mental stability, emotional capacity, tolerance to stress and frustration). In a test where 30 recorded interviews from 1972/1973 were scored by 30 psychologists, the inter-rater reliability for the assessment of psychosocial functioning was found to be high (r  =  0.86) [22]. If there was indication of severe mental problems, the patients were referred to a psychiatrist. The psychiatric diagnoses were classified according to International Classification of Disease, Revision 8 (ICD-8) [23].

In this study, we chose confounders, which have been shown to have significance for both morbidity and mortality based on literature and earlier results from this cohort.

The confounders were: own psychiatric problems (yes vs no), cognitive functioning (based on four intellectual and cognitive tests). The results of these tests were assessed on a nine-point scale with normal distribution. We divided the variable into three levels; below average group (1–3), average group (4–6) and above average group (7–9). Emotional control (very bad, bad vs. very good, good, medium), psychiatric diagnosis at conscription (yes at least one diagnosis vs. no diagnosis), conduct problems at school (yes at least once vs. no), previous contacts with police and/or juvenile authorities (yes, several or sometimes vs. no) problem drinking, (yes,≥210 g pure alcohol per week, having ever taken an ‘eye-opener’, being intoxicated often, having been taking into custody for public drunkenness on at least one occasion vs. no), and illicit drug use was coded (yes vs. no), with yes defined as used illicit drugs 10 times or more or taken drugs intravenously.

The conscription in Sweden was mandatory in 1969–1970 for all 18–20 years’ Swedish men, who were not relieved due to illness, medical or psychiatric reasons. Most participants completed the questionnaires, but some items in the questionnaires were not answered. The eight confounders included in the analyses had an internal non-participant-rate of 0.13–4.6% (Table 1).

In-patient and mortality data was linked at Statistics Sweden via the unique personal number for each subject in the cohort. This personal number was then replaced with an individual serial number making the data anonymous to the researchers, after approval of Regional ethical review board in Stockholm (Dnr 2007/174–31, Dnr 2008/1086–31/5).

### Statistical analyses

Since the study population is clearly defined (49,433 men who were compulsory conscripted to military service between 1 July in 1969 and 30 June in 1970) and no new study participants entered after that, the exposures (adolescent criminality registered between 15 and 18 years) and the potential confounders occurred before the conscription/were assessed during conscription, and we did not investigate any time-varying exposures; we used crude and multivariate Cox proportional regression analysis to calculate hazard ratios (HRs) with 95% confidence intervals (95% CI) for overall and cause specific mortality.

Confounders measured at conscription were used in adjusted analyses in relation to time to death and in-patient care. We calculated the surveillance time from 1 of January 1970 until death or until 31 December 2004 and 2006 for inpatient care records for all subjects in the cohort. The mean follow-up time was 20.1 years after taking into account the surveillance time for the deceased subjects. We censored for person time and mortality in Tables 2, 3. We did not censor for emigration in the calculation of person time due to the lack of such data. To compare the proportions of the outcome measures (diagnose for inpatient care stays and mortality) between the three criminal groups (violent, non-violent and the group with no criminality), we performed Likelihood ratio test by using proportional hazard regression analyses (PHREG), when we calculated chi2 and *p*-value. [24].

In the multivariate analyses, we included only significant variables from the earlier bivariate analyses in relation to the outcomes. We included only those subjects who had answered all the questions in the multivariate models.

We tested the proportional hazard assumption for each predictor (X) in crude and multivariate analyses by using a time-dependent explanatory variable in the model (X*(log time – average value of the log time). If the *P*-value was significant (*P* < .05), the proportional hazard assumption was not fulfilled and we excluded the variable from the analyses. We performed the statistical analyses by using the SAS statistical software 9.4 [25].

## Results

The results showed that 6% of the total cohort had committed crimes already at the time prior to conscription (18–20 years of age). Of these, 5.6% had committed non-violent and 0.4% violent crimes.

Nearly 81% of all death cases (*n* = 2671) had been treated at hospital for any diagnose compared to 19.3% in those still alive.

### Covariates

Table 1 shows eight covariates in relation to mortality and inpatient care. Ten percent of the death cases had reported problem drinking and 4% had no such problem at the time of conscription. Similarly, substance use (illicit drugs), the corresponding numbers were 8.8 and 5%, respectively. The majority of those persons, which have been treated at hospital, had also high percentage of alcohol and substance use.

**Table 1:** Distribution of psychological, behavioral and substance use in relation to mortality and in-patient care

Variables		Total cohort	Death cases		In patient care	
			n	%	n	%
Medication for own psychiatric problems	Yes	5531	501	9.06	4098	74.09
	No	42,665	2107	4.94	27,822	65.21
	Missing	638	63	9.87	455	71.32
Low intelligence	Yes	9298	730	7.85	6704	72.10
	No	39,461	1936	4.91	25,613	64.91
	Missing	75	5	6.67	58	77.33
Low emotional control	Yes	14,753	1120	7.59	10,500	71.17
	No	34,080	1551	4.55	21,875	64.19
	Missing	1	0	0	0	0
Psychiatric diagnosis at conscription	Yes	6081	600	9.87	4538	74.63
	No	41,877	2013	4.81	27,213	64.98
	Missing	876	58	6.62	624	71.23
Conduct problems at school	Yes	11,657	926	7.94	8498	72.90
	No	36,612	1689	4.61	23,471	64.11
	Missing	565	56	9.91	406	71.86
Previous contacts with police and/or juvenile authorities	Yes	13,769	1129	8.20	9939	72.18
	No	34,276	1478	4.31	21,877	63.83
	Missing	789	64	8.11	559	70.85
Problem drinking	Yes	6391	646	10.11	4798	75.07
	No	42,443	2025	4.77	27,577	64.97
	Missing	0	0	0	0	0
Substance use	Yes	5303	465	8.77	3771	71.11
	No	43,525	2206	5.07	28,598	65.70
	Missing	6	0	0	6	100

### Inpatient care

After taking into account follow-up time and mortality, hospitalizations due to alcohol and drug related diagnoses were significantly elevated for the violent group compared to non-violent criminals and no-criminals: 34.36, 23.69 and 5.33%, respectively (χ2 = 1033.23, *p* < .0001) (Table 2). The same pattern was found for hospitalization due to suicide attempt: 14.56, 8.56 and 2.10%, (χ2 = 228.41, *p* < .0001). Twice as many of either the violent- or non-violent men compared with non-criminal had been treated for at least one accident (Table 2).

**Table 2:** Adolescent criminality and hospital diagnoses^a^ Chi2, Sign^b^

	No crime (*n* = 45,899)	Non-violent (*n* = 2744)	Violent (*n* = 194)	Chi2, Sign.
Alcohol + Drug	5.33%	23.69%	34.36%	χ2 = 1033.23, p < .0001
Suicide attempt	2.10%	8.56%	14.36%	χ2 = 228.41, p < .0001
Accidents	21.79%	37.42%	37.95%	χ2 = 330.21, p < .0001
Circulatory disease	14.34%	18.30%	18.97%	χ2 = 48.85, *p* < .0001
Neoplasm	5.26%	6.24%	9.23%	χ2 = 15.63, p = .0004
Other diagnoses	29.07%	22.48%	21.54%	χ2 = 16.12, *p* < .0003
Any inpatient care stays	65.78%	79.09%	83.08%	χ2 = 156.45, p < .0001

^a^One subject can have several diagnoses

^b^Likelihood ratio test adjusted for time and mortality (PHREG)

**Fig. 1:**
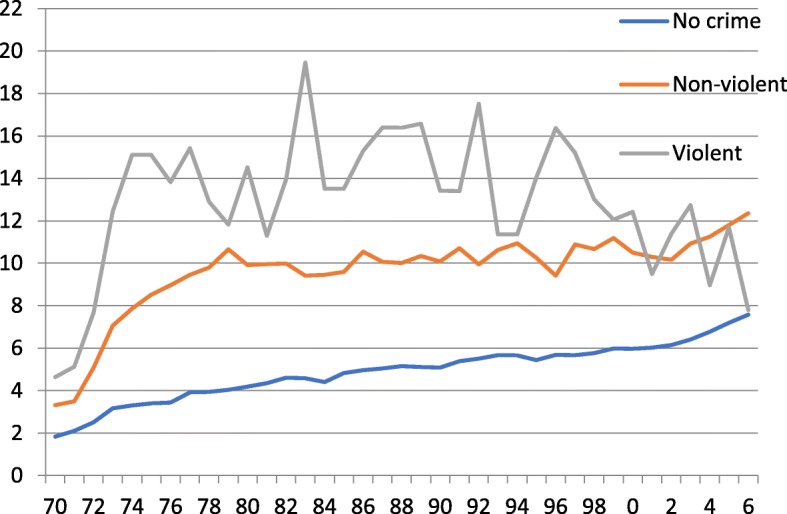
Incidence of in-patient care stays per living persons and years, 1970–2006

Figure 1 shows yearly incidence of inpatient care stays. The curves show a slightly increase for non-criminals from 2 to 10 per person and year over the years, while particularly violent offenders had a high incidence of admissions during 1980–1990 and then fell to a much lower level. The non-violent criminals have remained at about 20 during most of the observation period and a slight increase from 2003 up to 2006. It should be noted that the National Hospital Register reached nationwide coverage 1987.

### Mortality and causes of death

In Table 3, we show specific causes of death. Likelihood ratio test adjusted for person time and mortality was performed. Violent offenders had significantly higher mortality rate (21.13%) than non-violent offenders (12.90%) and non-criminals (4.96%) (χ2 = 296.33), *p* < .0001). Mortality due to suicide and drug-related diagnoses was significantly higher among violent offenders than the other two groups, while no significant difference was found for neoplasms. Nearly 3 % in both violent and non-violent criminals had died due to accidents, while 0.75% in the non-criminal group (χ2 = 139.29; *p* < .0001) (Table 3).

**Table 3:** Violent and non-violent offenders prior to conscription and causes of death. Chi2, Sign^a^

	No crime (*n* = 45,896)	Non-violent (*n* = 2744)	Violent (*n* = 194)	Chi2, Sign.
Suicide (*n* = 487)	0.93%	1.82%	5.15%	χ2 = 30.18, *p* < .0001
Alcohol and drug- related diagnosis (*n* = 378)	0.59%	3.35%	6.70%	χ2 = 159.31, *p* < .0001
Accident (*n* = 420)	0.75%	2.55%	2.58%	χ2 = 139.29, *p* < .0001
Circulatory disease (*n* = 400)	0.78%	1.49%	1.03%	χ2 = 15.52, *p* = .0004
Neoplasm (*n* = 560)	1.13%	1.35%	2.58%	χ2 = 4.95, *p* = .0843
Other cause (*n* = 426)	0.78%	2.33%	3.09%	χ2 = 32.90, *p* < .0001
All causes (*n* = 2671)	4.96%	12.89%	21.13%	χ2 = 296.33, *p* < .0001

^a^Likelihood ratio test adjusted for time and mortality (PHREG)

Crude and multivariate analyses for causes of death are presented in Table 4. In the crude analyses, violent offenders had more than 12 times higher risk for death due to an alcohol and drug related diagnosis compared to non-criminals. The corresponding figure for the non-violent criminals was decreased by half (HR = 5.94, 4.85–7.28). The corresponding figures for suicide were: HR = 6.84 and HR = 2.70, respectively. After adjustment for confounders (medication for own psychiatric problems, intelligence, emotional control psychiatric diagnosis at conscription, conduct problems at school, previous contacts with police and/or juvenile authorities, problem drinking and substance use), the hazards were still significantly elevated for both alcohol and drug related mortality and suicide and other diagnoses. The HR: s were elevated for the violent and non-violent offenders with no-offenders as reference category.

**Table 4:** Bivariate and multivariate analyses: Violent and non-violent offenders prior to conscription and causes of death

	Suicide	Alcohol and drug-related diagnosis	Accident	Circulatory disease	Neoplasm	Other diagnosis	Total death
	HR (crude)	HR (crude)	HR (crude)	HR (crude)	HR (crude)	HR (crude)	HR (crude)
Non-violent vs, no crime	2.70, 2.13–3.41	5.94, 4.85–7.28	4.46, 3.60–5.52	2.03, 1.47–2.83	1.29,0.93–1.80	1.50, 1.24–1.82	2.72, 2.43–3.04
Violent vs. no crime	6.84, 4.02–11.63	12.18, 7.49–19.80	4.96, 2.46–9.98	1.50, 0.37–6.01	2.57, 1.07–6.20	2.80, 1.33–5.89	4.69, 3.44–6.38
	HR adjusted^a^	HR adjusted^a^	HR adjusted^a^	Circulatory disease	Neoplasm	HR adjusted^a^	HR adjusted^a^
Non-violent vs, no crime	1.37, 1.03–1.81	1.79, 1.39–2.31	2.32, 1.75–3.07	1.85, 1.29–2.66	1.30, 0.91–1.86	1.55, 1.27–1.89	2,16, 1.93–2.42
Violent vs. no crime	2.14, 1.12–4.11	2.81, 1.66–4.77	2.54, 1.17–5.48	1.78, 0.44–7.13	3.10, 1.29–7.48	2.68, 1.20–5.99	4.29, 3.08–5.96

^a^Adjusted for medication for own psychiatric problems, intelligence, emotional control, psychiatric diagnosis at conscription, conduct problems at school, previous contacts with police and/or juvenile authorities, problem drinking and substance use

## Discussion

This study investigates adolescent criminality in relation to adult morbidity and mortality including admissions to hospital and causes of death. We analyzed effects of registered criminality before the conscription, which means that the person has been in contact with the correctional authorities and got a conviction, on adverse adult health outcomes and risk of death. Very few, less than 200 men had been convicted for a violent crime before the conscription, while non-violent crimes were 14-times more frequent in this population based cohort of Swedish men. The main finding was that violent criminals utilized more hospital care and had higher mortality rate than non-violent and non-criminals during the long-term follow-up. That was especially true for alcohol and drug related diagnoses for hospitalization and mortality as well as for suicide attempts and death by suicide. Why the violent offenders have poorer adult outcomes can be explained by many factors e.g. individual socio-economic risk factors, personality disorders, greater willingness to take risks and alcohol and substance abuse [26–31]. Starting at early ages with maladjusted behavior such as drug use in combination with criminality is associated with high mortality rate [16, 31, 32] as well as morbidity [33].

### Hospitalization

Suicide attempt, the strongest risk factor for suicide, was evident for one fifth of the total criminal group (violent, 13.92%, non-violent 8.35%) which was much higher than among non-criminals (2.04%). In total, the adolescent criminals accounted for 21% of the total number of suicide attempts in the cohort. A national case control study from Denmark reported that about one of three male suicide victims had a history of criminal court contacts and the risk of suicide was higher for those with frequent contacts and violent offending [33].

The results of this study show that almost 60% of the total criminal group had been hospitalized for an alcohol and/or drug diagnosis. This indicates that a very large proportion of criminals develop serious substance abuse, which in turn has serious health consequences including death. Therefore, it is important to screen for alcohol and drug use among young people with maladjusted behavior in order to prevent further substance use and crime.

It has been stated that adult criminals and especially violent criminals are frequently involved in accidents, mainly due to a combination of alcohol and impulsive behavior [34, 35]. In this study, we did not find any significant differences between violent and non-violent criminals regarding hospitalization because of accidents, but a much higher proportion compared to non-criminals.

When analyzing the yearly incidence of inpatient care stays, the curves are clearly higher for non-criminals and particularly for violent offenders during the most of the follow-up time. It should be noted that the National Hospital Register reached nationwide coverage 1987. The annual fluctuations of incidence of inpatient care among violent offenders are most probably due to small numbers getting even smaller at the end of the follow-up time due to mortality.

### Mortality and causes of death

The mortality rate during the observation-period was most elevated (21.1%) in violent offenders, followed by non-violent offenders (12.90%) compared to the non-criminals (4.96%). The causes of death like alcohol and drug related diagnoses and suicide was most prevalent in violent offenders, which shows the same risk pattern as for in-patient care diagnoses. The relationships between alcohol use, suicide and violent death have been studied by many researchers [1, 36, 37]. Lunetta et al., [37] found frequent alcohol abuse before the fatal event, especially among younger persons. However, the proportion of alcohol-related violent death varies widely between different studies [38, 39]. Likewise, in a recent study of mortality after self-poisoning among 1119 15–34 years young men and women, the risk of suicide was more than 60 times higher and all-cause mortality 26 times higher compared to the general population in the same age category [40].

Death due to accidents was much the same in both violent and non-violent criminals, but highly increased when comparing with the non-criminals. This indicates that a criminal life-style and risky behavior including alcohol and drug use entail higher risk for accidents and other cause of death [37–39]. In the multivariate analyses, we controlled for psychological and behavioral factors including substance use measured at the time of conscription, but the relationship between criminality, especially violent criminality, remained significant for alcohol and drug related diagnoses and suicide as well as accidents. The chosen variables have shown to be important for the outcomes both in prior studies based on this cohort and other scientific studies.

### Advantages and limitations

One of the advantages is the large national cohort with a long follow–up period of 37 years. Further, very few (2–3%) of the conscripts were excluded from the study mainly because of physical or psychiatric handicap. Another advantage was the many confounders, including social background, psychological and physiological factors collected at conscription (Table 1). The questionnaires were non-anonymous which could have affected the answers, with a lower response-rate on questions regarding criminality and drug use. A small number of the conscripts missed one or some of the items in the questionnaires. One could assume that these subjects had higher rates maladjusted behavior which could have affected the outcome [11]. However, we have no reason to believe that the risk for the outcomes (mortality and morbidity) has been considerable affected due the internal missing data. A compared of register and self-reported information for example alcohol and substance use and found good agreement between the different sources of data [21].

Another limitation of this study is that we do not have records of diseases that for example have only been treated in outpatient care. These diagnoses are not included in the In-patient care register. Some subjects could have experiences of many suicide attempts or non-fatal intoxications, and never been registered for these. The same applies for crimes, which do not come to the attention of the law enforcement authorities and thus remain unregistered. Further, it is difficult to balance confounder adjustment between under-adjustment (with residual confounding) and over-adjustment (with covariates lying directly on the causal pathway inadvertently included as confounders) and that, bearing this dilemma in mind, interpretation of the presented multivariable models ought to be carefully considered.

## Conclusions

Violent and non-violent crimes at young age are associated with multiple adverse outcomes and early death. Especially, mortality due to alcohol and drug use and suicide are prevalent causes of death. In addition, prevalence of suicide attempt is high in both criminal groups compared to non-criminals. That is also the case for hospitalization of alcohol and drug use and psychiatric care. Early prevention and identification of young people with destructive behavior is essential to stop or minimize negative future outcomes, which also entails great economic and social benefits for the society. It is important for health professionals to provide care to both victims as well as offenders in order to prevent hospitalizations and mortality [3].

## Abbreviations

CAP: child and adolescent psychiatric patients
CI: confidence interval
HR: Hazard ratio
ICD: International Classification of Diseases
